# Thioredoxin plays a key role in retinal neuropathy prior to endothelial damage in diabetic mice

**DOI:** 10.18632/oncotarget.18134

**Published:** 2017-05-24

**Authors:** Xiang Ren, Chen Li, Junli Liu, Chenghong Zhang, Yuzhen Fu, Nina Wang, Haiying Ma, Heyuan Lu, Hui Kong, Li Kong

**Affiliations:** ^1^ Department of Histology and Embryology, Dalian Medical University, Dalian 116044, Liaoning Province, China; ^2^ Department of Otorhinolaryngology, The Second Hospital of Dalian Medical University, Dalian 116023, Liaoning Province, China

**Keywords:** thioredoxin, diabetes, retina, apoptosis, sulforaphane

## Abstract

Diabetes is a chronic metabolic syndrome that results in changes in carbohydrate, lipid and protein metabolism. With diabetes for a long time, it increases the risk of diabetic retinopathy (DR) and long-term morbidity and mortality. Moreover, emerging evidence suggests that neuron damage occurs earlier than microvascular complications in DR patients, but the underlying mechanism is unclear. We investigated diabetes-induced retinal neuropathy and elucidated key molecular events to identify new therapeutic targets for the clinical treatment and prevention of DR. For *in vivo* studies, a high-fat diet and streptozotocin (STZ) injection were used to generate the diabetes model. Hematoxylin-eosin staining was used for morphological observations and measurements of the outer nuclear layer thickness. Electroretinography (ERG) was used to assess retinal function. For *in vitro* studies, Neuro2a cells were incubated in normal (5.5 mM) and high-glucose (30 mM) conditions. Flow cytometry assays were performed to analyze apoptosis. Additionally, real-time PCR and Western blotting analyses were carried out to determine gene and protein expression *in vitro* and *in vivo*. Taken together, the results indicated that retinal neuropathy occurred prior to endothelial damage induced by diabetes, and thioredoxin (Trx) plays a key role in this process. This underlying mechanism may be related to activation of the Trx/ASK1/p-p38/Trx-interacting protein pathway.

## INTRODUCTION

Diabetes mellitus (DM) is a chronic endocrine metabolic disorder that involves alterations in carbohydrate, fat and protein metabolism. DM is a major public health problem worldwide. In 2013, the International Diabetes Federation (IDF) estimated that over 8.3% of the adult population between ages 20 and 79 had diabetes, with 46% of these undiagnosed [1]. Approximately 350 million people worldwide have diabetes [1, 2], and 90-95% have type 2 DM (T2DM) [3]. DM can increase the risk of damage to tissues and organs, such as the retina, heart, and kidney [4], predominantly due to hyperglycemia.

Hyperglycemia promotes diabetic complications, such as diabetic retinopathy (DR). Retina damage induced by hyperglycemia is the primary cause of visual impairment in individuals worldwide and it occurs more frequently in individuals with poor glycemic control and with a long history of diabetes [5]. Other major risk factors for retinopathy include hypertension, renal disease [6], and dyslipoproteinemia [7, 8]. Retina damage is an ocular manifestation of DM that affects up to 80% of patients who have had DM for 10 years or longer [9, 10]. The pathology of retina damage is complex, and a better understanding of the underlying mechanisms of retina damage, which are currently incompletely understood, is important for the identification of novel treatments. In a previous clinical study [11, 12], retina damage was classified as a vascular disease characterized by endothelial cell proliferation and vascular permeability, which led to edema. A recent report found glial reactivity and reduced thickness of all retinal layers, suggesting that neuronal degeneration precedes the vascular changes in early-stage DR [13–15].

Under chronic hyperglycemic conditions, reducing sugars, such as glucose, and various proteins and lipids non-enzymatically react with free amino groups of proteins to form advanced glycation end products (AGEs) at an accelerated rate [16–18]. In several animal models of DM, increased concentrations of AGEs have been associated with various tissue and organ damage induced by diabetic conditions, such as nephropathy, DR, neuropathy, impaired dermal healing and age-related disease [19, 20]. Moreover, several studies have suggested that AGEs mediate the apoptosis observed during the pathogenesis of biophysical disorders [21]. Oxidative stress, which is considered the leading cause of retina damage, can be induced by hyperglycemia. Reactive oxygen species (ROS) are destructive products generated by oxidative stress, and excess ROS production can directly activate a downstream apoptotic pathway [22].

The thioredoxin (Trx) system, a redox system that includes Trx, thioredoxin reductase (TrxR) and NADPH, regulates the cellular redox balance [23]. Trx is a ubiquitously expressed small (12 kDa) dithiol protein that contains redox-active cysteine residues and plays a crucial role in redox regulation related to cell survival and growth [24]. As an antioxidant, Trx exerts its ROS-scavenging function in conjunction with thioredoxin peroxidase in the cytoplasm and nucleus [25]. Intravitreally injected Trx can relieve neural retina damage induced by excitatory amino acids, suggesting that Trx is neuroprotective in the retina [26].

Sulforaphane (SF) is a naturally occurring isothiocyanate compound isolated from cruciferous vegetables, such as broccoli and cabbage. Several studies have indicated that SF prevents diabetes-induced cardiac [27] and aortic damage [28], as well as testicular apoptotic cell death [29] and that SF is an efficient antioxidant against ROS-mediated injury [30]. SF activates nuclear factor erythroid 2-like 2 (Nrf2) to up-regulate cellular antioxidants, thus protecting against oxidative stress and damage [31], and attenuates high-fat diet (HFD)-induced visceral adiposity, adipocyte hypertrophy and lipid accumulation in the liver [32]. We previously showed that SF prevents retinal photoreceptor cell degeneration in the homozygous tubby mutant mouse by up-regulating the Trx system [33].

Based on previous findings, we used Neuro2a cells [34] and an STZ/HFD (high-fat diet)-induced mouse model to investigate the protective effects of Trx against retinal neuropathy prior to endothelial damage and to identify the related mechanisms *in vitro* and *in vivo* to provide evidence for new clinical therapeutic targets of DR.

## RESULTS

### Retinal neuropathy prior to endothelial damage in diabetic mice

**Figure 1:**
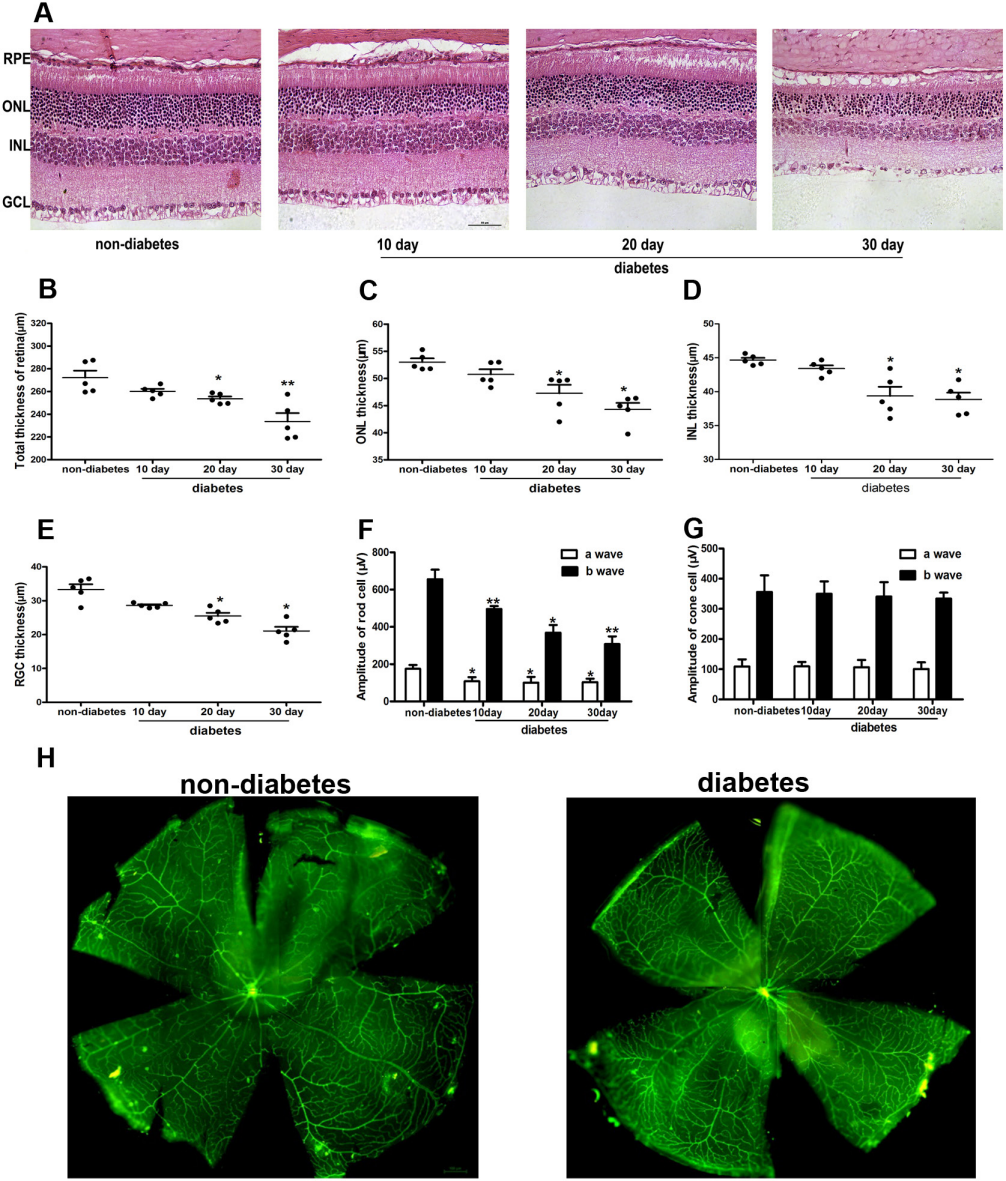
Morphological analysis of neuronal cells and vessels in the retina of diabetic mice **(A)** Morphological analysis of retina neuronal cells by H&E staining in diabetic mice. The thickness of the whole retina **(B)**, ONL **(C)**, INL **(D)**, and RGC layer **(E)** in diabetic mice. **(F)-(G)** The functional analysis of the retina by ERG in diabetic mice. **(H)** Whole-mount retinas labeled with lectin-BS in diabetic mice. The data are presented as the mean ± SD. (n=5 in each group) * p<0.05, * * p<0.01.

To assess the effects of diabetes on retinal neuronal cells, we fed mice a HFD and injected them with STZ to generate the DM model. After 10, 20, and 30 d, we isolated the mouse retinas and used H&E staining to observe the retinal morphology. As shown in Figure 1A, retina thickness was gradually reduced in the diabetic mice at 10, 20, and 30 d compared with that of the non-diabetic mice. The retina thickness was defined as the distance from the retinal pigment epithelium layer (RPE) to the ganglion cell layer (GCL). Moreover, the thicknesses were reduced around 10% in the whole retina, ONL, INL and RGC layer in diabetic mice at 10, 20, and 30 d (Figure 1B-1E) (P<0.05). Additionally, we measured the retina thickness in the non-diabetic mice at 10, 20, and 30 d. There were no significant differences (data was not shown).

Simultaneously, we used ERG to assess the function of the retina, including rod cells and cone cells. The results indicated that the a- and b-waves of rod cells were decreased about 50% in diabetic mice compared with those of non-diabetic mice (P<0.05). However, the a- and b-waves of cone cells were normal (Figure 1F, 1G). Furthermore, we labeled whole-mount retinas with lectin-BS, and no vessel damage was observed in the diabetic mice compared with that of the non-diabetic mice (Figure 1H).

### The effect of Trx on retina neuronal cell degeneration and related mechanism in diabetic mice

**Figure 2:**
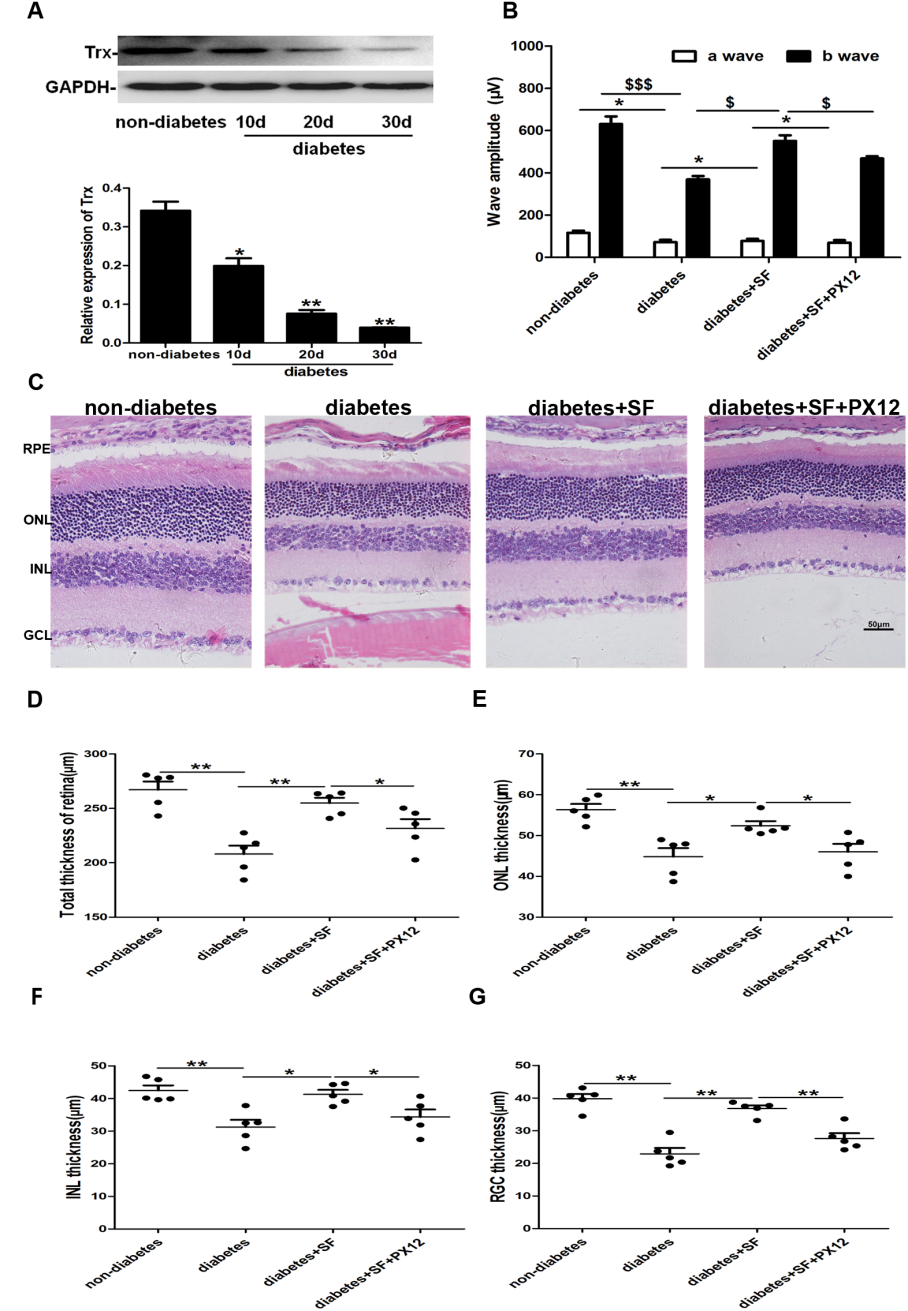
The relationship between Trx and retina neuronal cell degeneration in diabetic mice **(A)** Trx expression in the retinas of diabetic mice. **(B)-(C)** Functional and morphological analysis of the protective effect of Trx on retina neuronal cell degeneration by H&E staining and ERG. The thickness of the whole retina **(D)**, ONL **(E)**, INL **(F)**, and RGC layer **(G)** in diabetic mice. The data are presented as the mean ± SD (n=5 in each group). * p<0.05, * * p<0.01, $ $ $ p<0.001, $ p<0.05.

Trx expression was decreased gradually in diabetic mice at 10, 20, and 30 d (decreasing about 70%) (Figure 2A) (P<0.05). The data indicated that Trx expression decreasing could be related to retina neuronal cell degeneration in diabetic mice.

Diabetic mice were treated with SF, which up-regulates Trx expression, and PX12, a Trx inhibitor, to confirm the role of Trx in the retina neuronal cell degeneration. To determine the role of Trx in this process, we divided the mice into four experimental groups: non-diabetes, diabetes, diabetes+SF, and diabetes+SF+PX12. The morphological analysis showed that the total thicknesses of the retina, ONL, INL, and RGC layer were decreased in diabetic mice compared with those of the non-diabetic mice, but retinal thickness decreasing speed was reduced after SF treatment (P<0.05). However, these parameters were decreased in the diabetes+SF+PX12 group compared with those in the diabetes+SF group (Figure 2C-2G) (P<0.05). The ERG analysis data showed the a- and b-waves were lower in the diabetes group compared with those in the non-diabetes group, and they increased after SF treatment (P<0.05). However, the a- and b-waves were lower in the diabetes+SF+PX12 group compared with those of the diabetes+SF group (Figure 2B) (P<0.05). These results indicated that Trx played an important role in delaying retina neuronal cell degeneration in diabetic mice.

**Figure 3:**
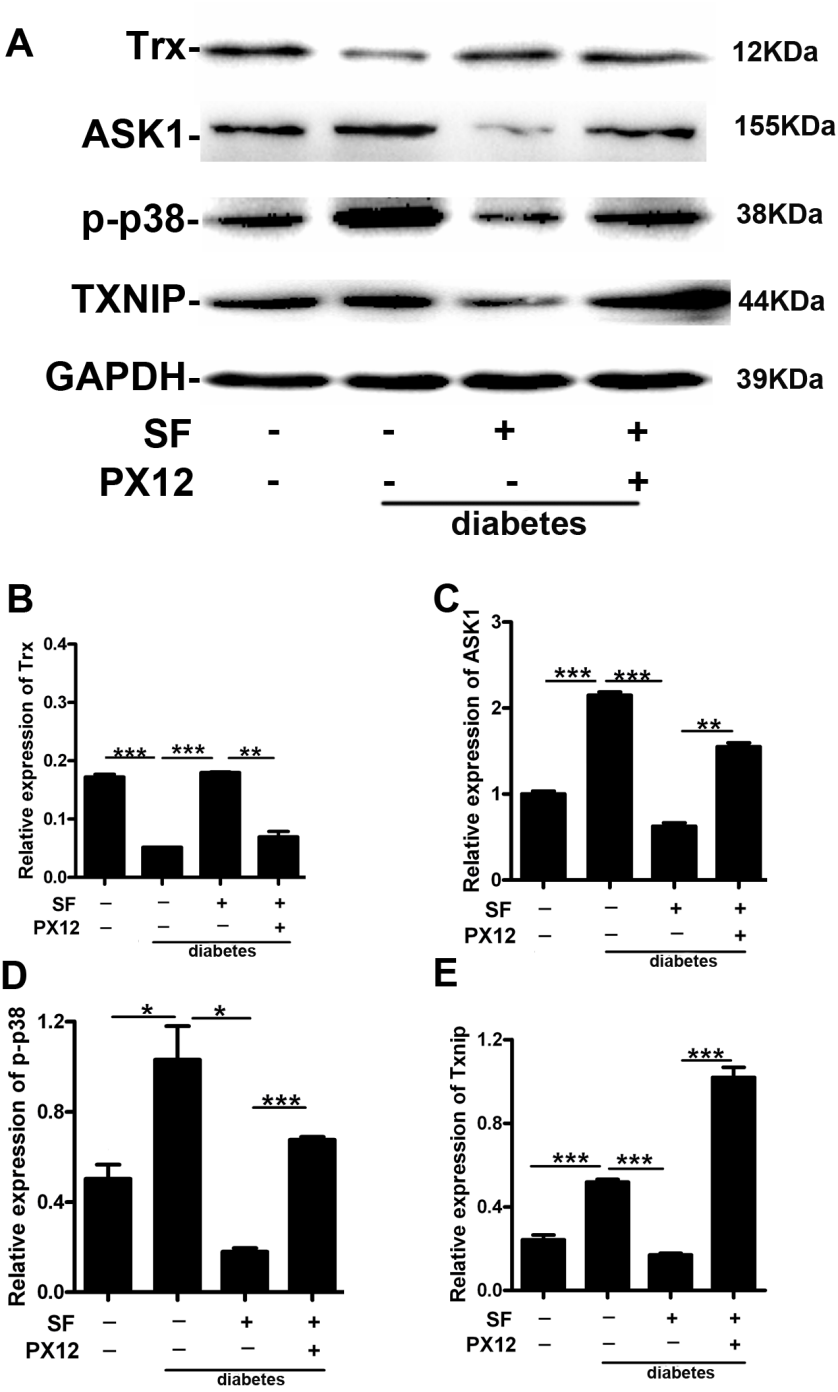
The mechanism by which Trx delays retina neuronal cell degeneration in diabetic mice Expression of **(A)-(B)** Trx, **(A)-(C)** ASK1, **(A)-(D)** p-p38, and **(A)-(E)** Txnip. The data are presented as the mean ± SD (n=5 in each group). * p<0.05, * * p<0.01, * * * p<0.001.

*In vivo*, we performed additional studies to further elucidate the related mechanisms. In the animal model, Trx protein expression decreased, and ASK1, p-p38 and Txnip protein expression was up-regulated in the diabetes group compared with that in the non-diabetes group. However, SF increased Trx expression and decreased ASK1, p-p38 and Txnip expression (diabetes+SF group versus diabetes group). Compared with the diabetes+SF group, Trx expression was decreased, and ASK1, p-p38 and Txnip were up-regulated in the diabetes+SF+PX12 group (Figure 3A-3E) (P<0.05).

### The effect of Trx on neuropathy prior to endothelial damage induced by high glucose (HG) *in vitro*

**Figure 4:**
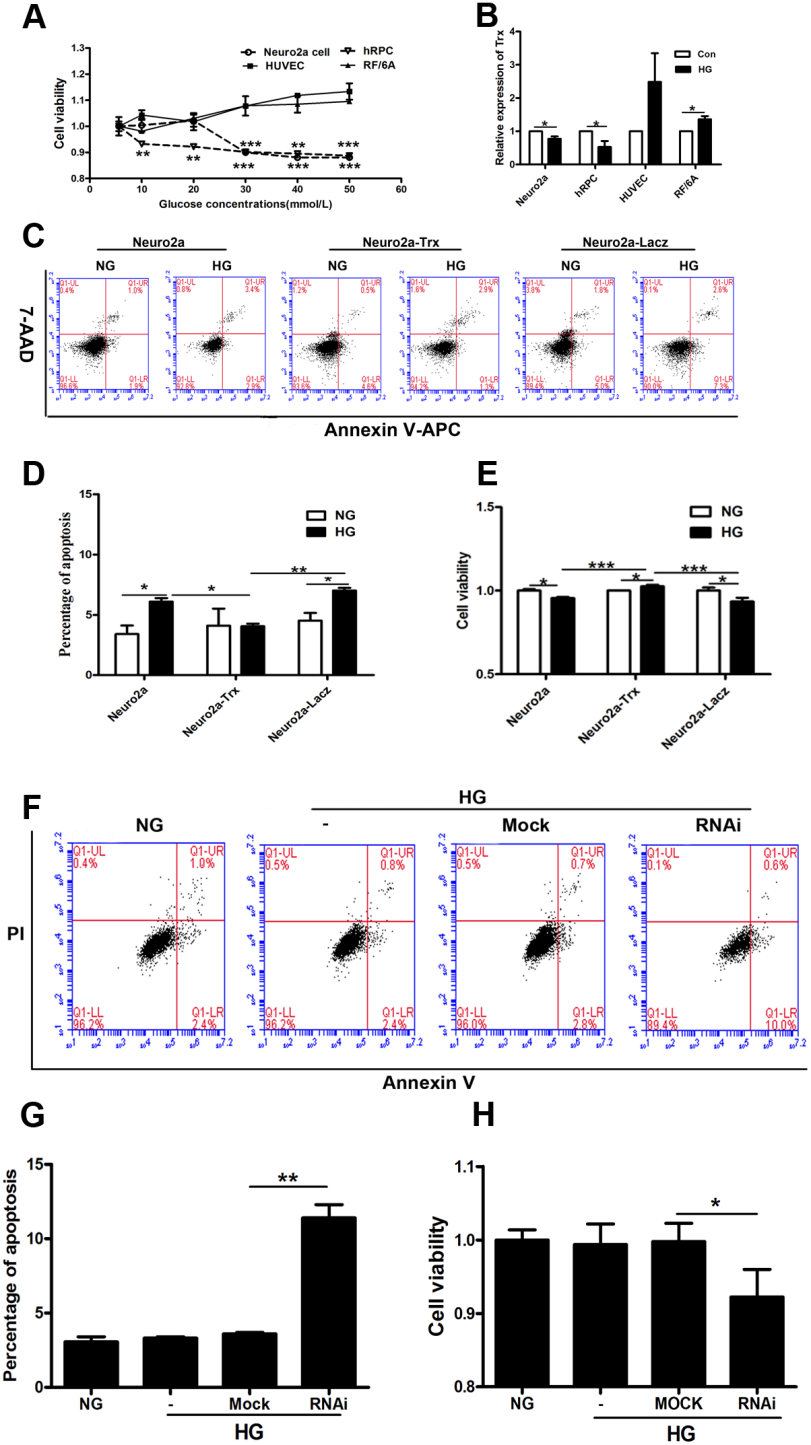
The effect of Trx on neuropathy prior to endothelial damage induced by hyperglycemia *in vitro* **(A)** The viability of Neuro2a cells, hRPCs, RF/6A cells, and HUVECs was measured by CCK8 assays after treatment with different HG concentrations for 24 h. **(B)** Trx expression was detected by real-time PCR in Neuro2a cells, hRPCs, RF/6A cells, and HUVECs after treatment with/without HG (30 mM). **(C)-(D)** Apoptosis analysis of Neuro2a, Neuro2a-Trx, and Neuro2a-LacZ cells after treatment with/without HG (30 mM). X axis is Annexin V-APC staining and Y axis is 7-AAD staining. **(E)** Neuro2a, Neuro2a-Trx, and Neuro2a-LacZ cell viability after treatment with/without HG (30 mM). **(F)-(G)** The effect of Trx on apoptosis of HUVECs with/without HG treatment (30 mM). X axis is Annexin V staining and Y axis is propidium iodide (PI) staining. **(H)** The effect of Trx on cell viability of HUVECs with/without HG treatment (30 mM). The data are presented as the mean ± SD (n=3 in each group). * p<0.05, * * p<0.01, * * * p<0.001.

To confirm the role of Trx in this process, we used Neuro2a cells, hRPCs, RF/6A cells, and HUVECs for *in vitro* studies. The cells were treated with different HG concentrations. The viability of Neuro2a cells and hRPCs decreased from 30 mM to 50 mM glucose compared with that of the control treatment (normal glucose, NG) (P<0.05). However, HUVEC and RF/6A cell viabilities were unchanged compared with that of the control group (Figure 4A) (P>0.05). Moreover, we detected Trx expression in HUVECs, RF/6A cells, Neuro2a cells and hRPCs after HG treatment; Trx expression was decreased in hRPCs and Neuro2a cells compared with those of the control groups (P<0.05). However, Trx expression was up-regulated in RF/6A cells (P<0.05) and was unchanged in HUVECs (P>0.05) compared with that of the control groups (Figure 4B). These data indicated that Trx expression could be related to this process that neuropathy prior to endothelial damage induced by HG.

Thus, apoptosis was analyzed in Neuro2a, Neuro2a-lacZ, and Neuro2a-Trx cells by flow cytometry and CCK8 assays. As shown in Figure 4C-4E, the percentage of apoptotic Neuro2a and Neuro2a-lacZ cells increased in the HG groups compared with that of the normal control groups (P<0.05). However, the percentage of apoptotic Neuro2a-Trx cells was not different in the HG group compared with the normal control group (P>0.05). Moreover, Neuro2a and Neuro2a-lacZ cell viability decreased in the HG groups compared with the normal control groups (P<0.05), but Neuro2a-Trx cell viability increased in the HG group compared with the normal control group (P<0.05).

Moreover, we used the RNAi method to knock down the expression of Trx in HUVECs and then performed apoptosis analysis using flow cytometry and CCK8 assays. As shown in Figure 4F-4H, the percentage of apoptotic HUVECs was unchanged in the HG groups compared with that in the normal control groups (P>0.05). However, the percentage of apoptotic HUVECs was increased after silencing of Trx (P<0.05). Moreover, HUVEC viability was unchanged in the HG groups compared with that of the normal control groups (P>0.05), but it was decreased after silencing Trx (P<0.05).

**Figure 5:**
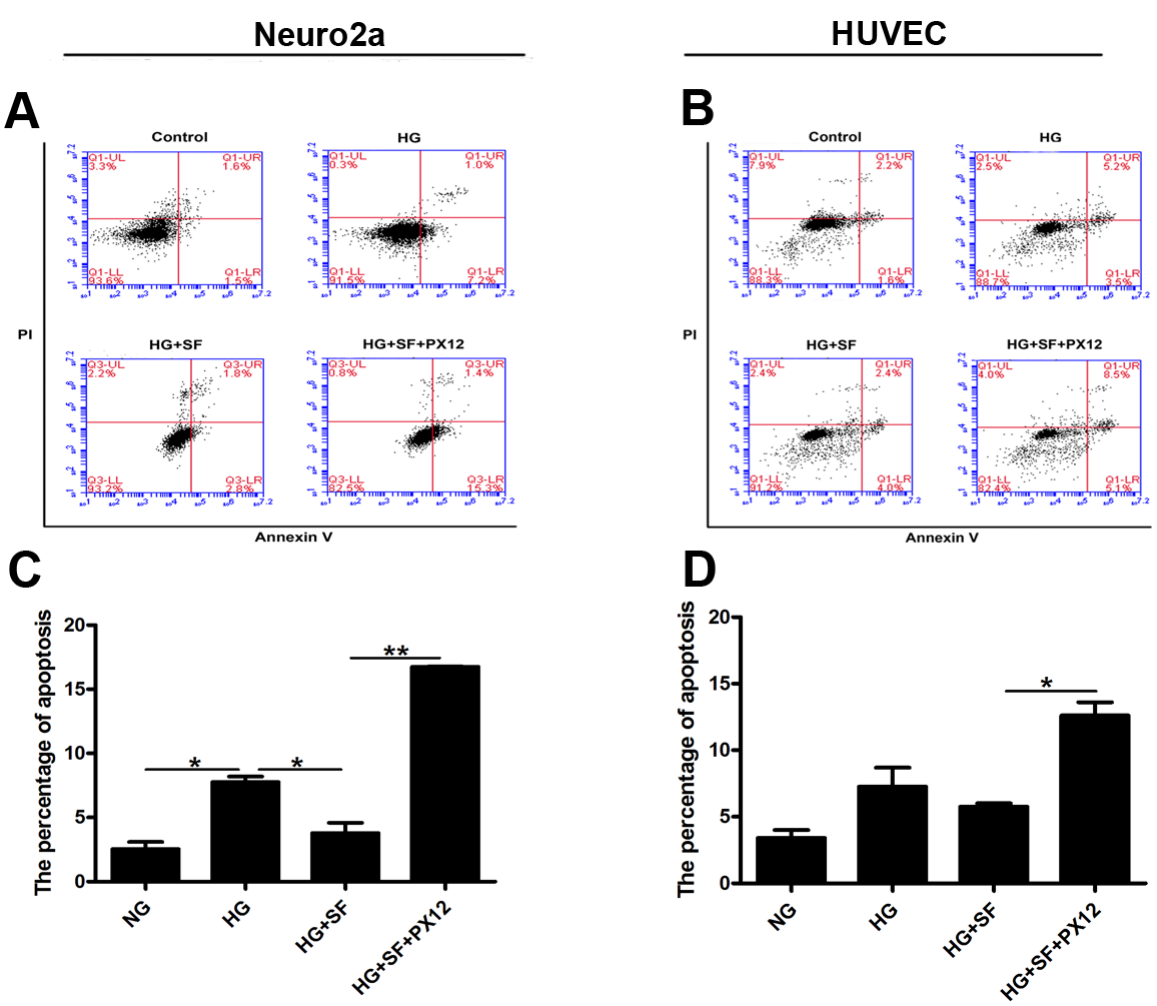
Effect of Trx on the apoptosis of Neuro2a cells and HUVECs **(A)-(C)** Apoptosis analysis of Neuro2a cells with/without SF or PX12 treatment in HG conditions. X axis is Annexin V staining and Y axis is propidium iodide (PI) staining. **(B)-(D)** Apoptosis analysis of HUVECs with/without SF or PX12 treatment in HG conditions. X axis is Annexin V staining and Y axis is propidium iodide (PI) staining. The data are presented as the mean ± SD (n=3 in each group). * p<0.05, * * p<0.01, * * * p<0.001.

We treated Neuro2a cells with or without SF and/or PX12 to confirm the role of Trx in hyperglycemia-induced neuropathy *in vitro*. In the apoptosis analysis, the percentage of apoptotic Neuro2a cells increased after HG treatment compared with that of the control treatment; furthermore, SF, which up-regulates Trx expression, decreased the percentage of apoptotic cells induced by HG compared with the HG-only group (P<0.05). However, when Trx expression was inhibited by PX12, the percentage of apoptotic cells was higher in the SF- and PX12-treated group compared with that of the SF-treated group under HG conditions (Figure 5A, 5C) (P<0.05). Moreover, we treated HUVECs with or without SF and/or PX12. In the apoptosis analysis, HG treatment affected the percentage of apoptotic HUVECs compared with that of the control treatment, but the difference was not significant (P>0.05); furthermore, SF, which up-regulates Trx expression, decreased the percentage of apoptotic cells induced by HG compared with that of the HG-only group (P<0.05). However, when Trx expression was inhibited by PX12 under HG conditions, the percentage of apoptotic cells was higher in the SF- and PX12-treated group compared with that of the SF-treated group (Figure 5B, 5D) (P<0.05). The data indicated that Trx played a key role in hyperglycemia-induced neuropathy prior to endothelial damage.

### The mechanism by which Trx affects retina neuronal cell degeneration in diabetic mice and HG-induced changes

The above studies indicated that Trx played an important role in delaying retina neuronal cell degeneration in diabetic mice. However, the related mechanism was unclear. Thus, we detected certain proteins by western blot to ascertain the mechanism and to determine why photoreceptor degeneration occurred earlier than microvascular changes in a HG environment.

**Figure 6:**
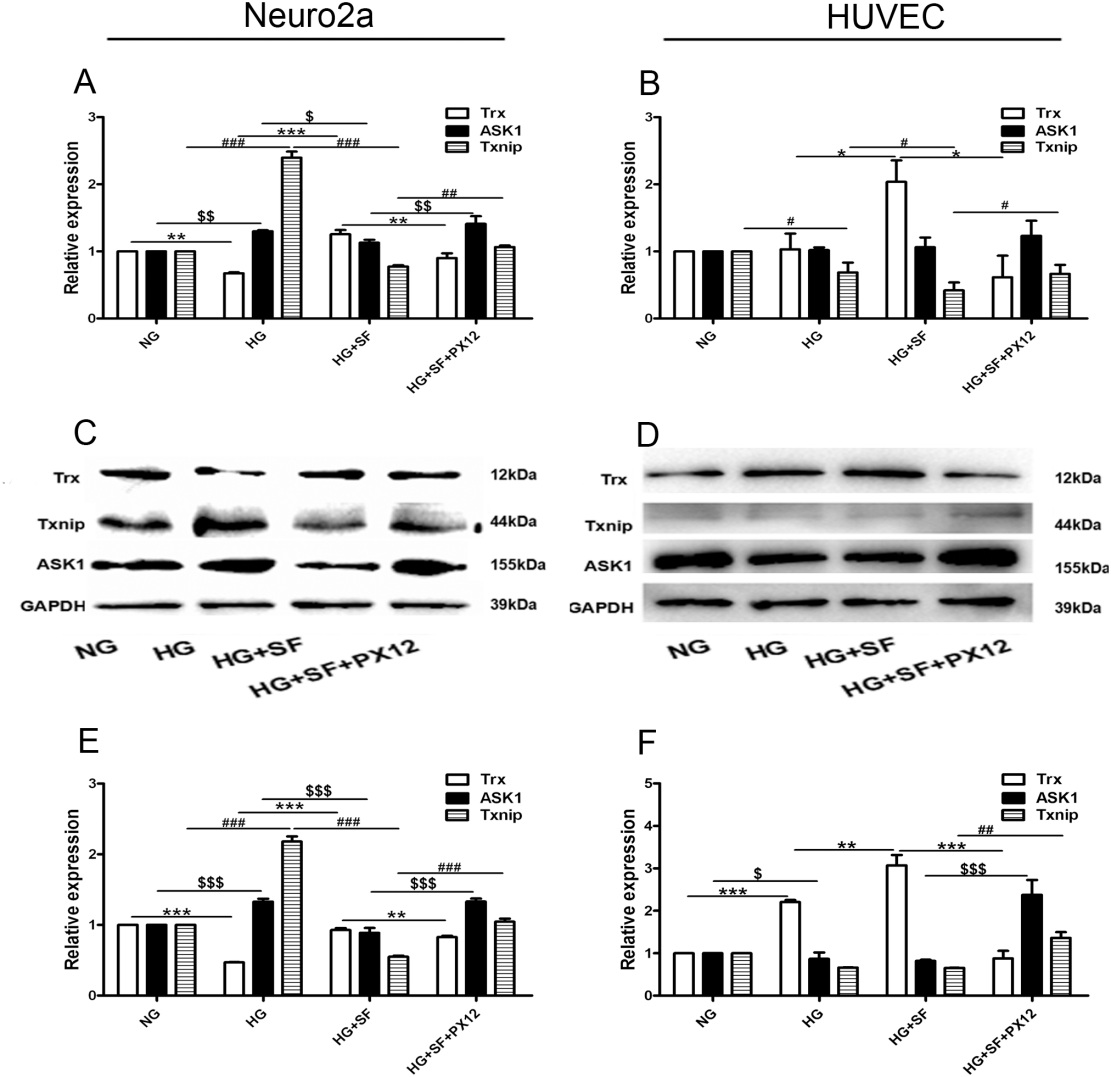
The mechanism by which Trx delays neuropathy under HG conditions at the mRNA and protein levels *in vitro* **(A)** Trx, Txnip, and ASK1 mRNA expression in Neuro2a cells. **(C)-(E)** Trx, Txnip, and ASK1 protein expression in Neuro2a cells. **(B)** Trx, Txnip, and ASK1 mRNA expression in HUVECs. **(D)-(F)** Trx, Txnip, and ASK1 protein expression in HUVECs. The data are presented as the mean ± SD (n=3 in each group). * p<0.05, * * p<0.01, * * * p<0.001. $ p<0.05, $$ p<0.01, $$$ p<0.001. # p<0.05, ## p<0.01, ### p<0.001.

In cell culture, Trx expression decreased at the mRNA and protein level in the HG group compared with the NG group, and both ASK1 and Txnip were up-regulated in the HG group compared with the NG group (P<0.05). However, in the HG+SF group compared with the HG group, Trx expression was increased, and ASK1 and Txnip expression was decreased (P<0.05). We also determined that Trx expression was down-regulated and that ASK1 and Txnip were up-regulated in the HG+SF+PX12 group compared with the HG+SF group (Figure 6A, 6C, 6E) (P<0.05). Moreover, Trx, ASK1, and Txnip mRNA expression was detected in HUVECs. In the HG group compared with the NG group, Trx mRNA expression was not changed (P>0.05), whereas Txnip was down-regulated. However, in the HG+SF group compared with the HG group, Trx expression was increased, and Txnip expression was decreased. In HUVECs, Trx protein expression was up-regulated by HG (HG group versus NG group) and SF (HG+SF group versus HG group) and was down-regulated by PX12 (HG+SF+PX12 group versus HG+SF group). Txnip expression was not changed in either the HG group or the HG+SF group (P>0.05), but it was up-regulated in the HG+SF+PX12 group compared with the HG+SF group (P<0.05). Moreover, ASK1 expression was lower in the HG group compared with the NG group, higher in the HG+SF+PX12 group compared with the HG+SF group, and unchanged in the HG+SF group compared with the HG group (Figure 6B, 6D, 6F) (P<0.05). These data could explain the mechanism underlying why retina neuronal cell degeneration occurred earlier than microvascular changes in a HG environment.

## DISCUSSION

Hyperglycemia is one of the main characteristics of DM. It can damage many organs, such as the retina, kidney, brain, and heart. DR is one of the complications of diabetes. In the past, DR was considered a vascular disease, suggesting endothelium cell damage in a HG environment. However, in our research, we found that neuronal damage occurred before vascular damage under HG conditions *in vitro* and *in vivo* (Figures 1, 4 and 5). There are some other research also reported that in db/db mice the vascular damage will be happening around 3 months [35]. In our research, Trx expression different maybe is the reason of neuronal damage occurred before vascular damage in diabetes. Moreover, damage within the photoreceptor cells occurred in rod cells, not cone cells. The major reason for this phenomenon is rod-derived cone viability factor (RdCVF), which increases cone cell survival [36, 37]. Based on these findings, it became apparent that understanding the related mechanism and identifying the key molecules were important.

Previous studies identified oxidative stress, which can induce apoptosis associated with diabetes, as the primary disruptor of sulfur-based redox systems. Trx is an endogenous disulfide reductase and thioreductase that is involved in the intracellular redox process. The active fragment of Trx, CGPC, changes the redox status of disulfide and disulfide compounds, which provides an electron-based system to perform a variety of antioxidant enzymatic reactions [38]. Trx, which has general intracellular antioxidant activity, protects against oxidative stress [39]. Some studies have shown that the Trx system plays an important role in protecting the function of islet cells. STZ-induced diabetes and islet β cell destruction can be prevented by high Trx expression [40]. As shown in Figures 3 and 5, Trx expression decreased under HG conditions *in vitro* and *in vivo*, indicating that Trx is likely a key molecule in protecting photoreceptor cells from HG-induced degeneration. However, Trx expression was up-regulated in HUVECs and RF/6A cells. Other studies [41, [42] have reported that RF/6A cell viability increases at glucose concentrations of 5 mM to 50 mM and that a HG environment increases the proliferation of HUVECs. Trx increases the proliferation of many cell types in various environments, and our experimental conditions (HG) were suitable for cell growth; therefore, Trx expression was up-regulated after HG treatment. These findings indicated that Trx likely played a key role in photoreceptor cell degeneration prior to endothelial damage under HG conditions.

SF can promote retinal photoreceptor cell growth and up-regulate Trx expression [33]. In our research, we found that SF up-regulated Trx expression to inhibit photoreceptor cell damage *in vitro* and *in vivo*. We concluded that Trx is important for protecting retinal photoreceptor cells. Moreover, excessive ROS levels are a key factor [43] in islet β cell damage induced by oxidative stress. ROS also participate in a series of intracellular second messenger-activated signaling pathways, including those that regulate glucose metabolism, thereby leading to increased gluconeogenesis in fat, skeletal muscle and liver tissue; therefore, increased oxidative stress exacerbates the pathological consequences of diabetes [44]. In our previous study, we found that up-regulating Trx expression decreased ROS generation [45], and several reports have confirmed that SF prevents the activation of HG-induced downstream ROS pathways and eliminates ROS to protect islet β cells. Furthermore, SF suppresses the excessive secretion of insulin in response to HG stimulation, thereby reducing obesity and insulin resistance [46]. Therefore, we hypothesized that SF prevented retina photoreceptor cell damage by up-regulating Trx expression and thus decreasing ROS generation.

The mitogen-activated protein kinase (MAPK) signal transduction pathway consists of three subfamilies: c-Jun N-terminal kinase (JNK), extracellular signal-related kinase (ERK) 1/2, and p38. The MAPK pathway is known to be activated by oxidative stress. The disruption of MAPK signaling pathways by oxidative stress can promote the development of human diseases, including various neurodegenerative diseases, DM, and cancer [47]. ASK1, a member of the MAPK kinase kinase (MKKK) family, functions by activating the JNK and p38 signaling pathways, which lead to stress-induced apoptosis and inflammation [48, 49]. When Trx is in a redox state, it binds to ASK1 and inhibits its activation. When Trx is oxidized in response to ROS or other stresses, it releases ASK1 and activates apoptosis signaling factors, including p38. Previous studies have shown that hyperglycemia induces the up-regulation of Txnip via the p38 signaling pathway and reduces the ROS-scavenging ability of Trx, thus increasing ASK1 levels, activating the p38 signaling pathway and causing irreversible damage [50]. Trx regulates the activity of ASK1, which is a major regulator of apoptosis. Trx is oxidized by ROS, which leads to ASK1 activation and promotes JNK and p38 phosphorylation, leading to apoptosis. The serine-threonine kinase p38 is an important signaling molecule that regulates many cellular functions in response to stress. Li and others have shown that inhibition of the p38 signaling pathway may be diminished by the HG-induced production of ROS, which up-regulate Txnip expression and reverse the redox effects of Trx [51]. Therefore, reducing Txnip expression may be an effective strategy for protecting islet β cells from damage under conditions of T1DM or T2DM [52]. The study by Saitoh showed that Trx expression inhibits ASK1 activity and that suppressing Trx increases ASK1 activity. These findings indicate that Trx may act as a functional inhibitor of ASK1 [53].

Txnip, also referred to as vitamin D3 up-regulating protein 1 (VDUP-1), inhibits the function and activity of Trx [54], and endogenous Txnip acts as a Trx inhibitor in cells [55]. Txnip is up-regulated by glucose in patients with diabetes and in animal models of diabetes, and it plays a critical role in glucotoxicity, inflammation, and β cell apoptosis. Additionally, we found that Txnip deficiency protects against diabetes [56]. Chen [57], Shalev [58] and Corbett [59] reported similar results. Txnip can induce apoptosis in certain contexts, such as in the T2D rat model [60].

After SF and/or PX12 treatment, we detected specific molecules *in vitro* and *in vivo* to elucidate the mechanism of diabetes-induced retina neuronal cell damage and to determine why neuropathy occurred earlier than microvascular damage. Trx expression decreased under HG conditions, increased in response to SF treatment and then decreased after PX12 treatment *in vitro* and *in vivo*. The expression of ASK1, Txnip, and p-p38 increased under HG conditions, decreased in response to SF treatment and increased after PX12 treatment. However, in HUVECs, Trx expression increased in the HG group, increased in response to SF treatment and then decreased after PX12 treatment. ASK1 and Txnip expression decreased or did not change under HG conditions, decreased in response to SF treatment and increased after PX12 treatment. The results also showed that SF up-regulated Trx, which inhibited ASK1 and downstream p38 activity, thereby decreasing Txnip expression and inhibiting photoreceptor cell apoptosis. This phenomenon could explain why diabetes-induced photoreceptor cell degeneration occurred prior to endothelial damage.

In summary, our data concerning the effects of SF and PX12 show that Trx plays an important role in protecting against HG-induced neuronal damage *in vitro* and *in vivo*. Txnip, an endogenous molecule that is up-regulated by oxidative stress caused by HG, is a key factor involved in the induction of photoreceptor cell damage. Txnip expression is higher in HG environments; Txnip binds to Trx, leading to the activation of ASK1/p38 and downstream signaling pathways, thereby inducing apoptosis. The increase in p38 activity up-regulates the expression of Txnip, leading to apoptosis. SF can up-regulate Trx expression *in vivo* and *in vitro*. In turn, Trx inhibits the activity of ASK1 and Txnip to inhibit the p38 pathway, thus preventing HG-induced photoreceptor cell damage *in vitro* and *in vivo*.

However, the HG environment is complex, and many factors, including AGEs, may be key to protecting against neuropathy due to hyperglycemia-induced damage.

## MATERIALS AND METHODS

### Cell culture and reagents

Neuro2a cells, RF/6A cells, and HUVECs obtained from the Institute of Biochemistry and Cell Biology, Chinese Academy of Sciences (Shanghai, China), and hRPCs (human retinal progenitor cells) obtained from the He University in Liaoning were cultured in MEM(Gibco), RPMI 1640(Gibco), DMEM(Gibco), and advanced DMEM(Gibco), respectively. The medium was supplemented with 10% fetal bovine serum (FBS, Gibco), and the cells were cultured at 37°C in 5% CO_2_. The medium was replaced every 1 or 2 days. The cells were washed with PBS before the experiments. PX12 (TOCRIS Bioscience) was dissolved in 1 mM dimethyl sulfoxide (DMSO, Sigma) and stored at -20°C. The HG medium (200 mM) was stored at 4°C.

### CCK8 assay

Cells (Neuro2a cells, RF/6A cells, HUVECs and hRPCs) in the logarithmic growth phase were collected, trypsinized, pipetted into a single-cell suspension at a density of 3 × 10^4^ cells/ml, and then seeded in 96-well plates. The total volume of each well was 100 μl, and the plates were placed at 37°C in a 5% CO_2_ incubator. Cells were treated with or without HG for 24h when they reached the logarithmic growth phase. Then, 10 μl of CCK8 regent (Dojindo, Japan) was added to each well, and the cells were incubated at 37°C for 1 h; the absorbance at 450 nm was measured using a microplate reader.

### Flow cytometry analysis

Cells (Neuro2a cells and HUVECs) were washed twice with PBS before the experiments. The cells were collected via centrifugation at 1000 rpm for 5 min and then stained with Annexin V/propidium iodide (PI), Annexin V-APC/7-AAD and binding buffer (KeyGEN Biotech) at room temperature for 15 min. After mixing, the samples were analyzed using a flow cytometer. The data was analyzed by soft of BD Accuri C6.

### Western blot

Whole cell extracts (50 μg of protein/lane) were separated via SDS–PAGE, and the proteins were transferred to PVDF membranes (Millipore, Billerica, MA). The membranes were blocked for 1 h at room temperature in 5% non-fat milk and then incubated overnight at 4°C. The following rabbit primary antibodies were used: anti-p-p38 (Cell Signaling Technology, CST), anti-apoptosis signal-regulating kinase 1 (ASK1; CST), anti-Trx (CST), and anti-Trx-interacting protein (Txnip; Proteintech). A mouse primary antibody against anti-β-Actin (Santa Cruz Biotechnology, CA) was also used. The membranes were washed three times (10 min/wash) with Tris-buffered saline containing 0.1% Tween 20 (1X TTBS). Subsequently, the membranes were incubated in goat anti-rabbit IgG and goat anti-mouse IgG for 1 h at room temperature and washed 3 times (15 min/wash) with 1X TTBS. The membranes were visualized using an enhanced chemiluminescence system and X-ray film. The band intensities were measured using LabWorks 4.5. All of the primary and secondary antibodies were diluted in 1X TTBS and 2.5% non-fat dry milk.

### Quantitative real-time PCR

Total RNA was obtained from each cell preparation using TRIzol (Invitrogen). Reverse transcription was performed using a Perfect for Real-Time PCR Kit (Takara). Real-time PCR was conducted to measure Txnip expression using a SYBR-Green mixture (Takara) and reverse-transcribed cDNA as the template. PCR was performed in a final volume of 20 μl using an ABI Prism 7000 Sequence Detection System under the following conditions: 95°C for 30 s, followed by 40 cycles of 95°C for 3 s, 72°C for 30 s, and 55°C for 30 s. GAPDH was used as an internal control. The following primers were used: Txnip (human), forward: 5′-GAGTGTGGGTCCACCTTAGC-3′, reverse: 5′-TGTATCACAACATGGGCGCT-3′; Trx (human), forward: 5′-GGTGAAGCAGATCGAGAGCA-3′, reverse: 5′-CCACGTGGCTGAGAAGTCAA-3′; ASK1 (human), forward: 5′-TGACCATGAGGAACAGCCTTC-3′, reverse: 5′-GGTGAGCACTCTGGGAATCA-3′; and Trx (mouse), forward: 5′-CAAATGCATGCCGACCTTCCAGTT-3′, reverse: 5′-TGGCAGTTGGGTATAGACTCTCCA-3′. The primer sequences for GAPDH (mouse) and Txnip (mouse) were previously reported [61].

### Animal care

All the experimental procedures were conducted in accordance with institutional guidelines for the care and use of laboratory animals, and protocols were approved by the Institutional Animal Care and Use Committees of Dalian Medical University Laboratory Animal Center. Six-week-old male inbred BALB/c mice weighing 20-25 g (Dalian Medical University Laboratory Animal Center) were housed 6 per cage in an animal colony facility for 2 weeks. The animals were maintained in a room with a constant temperature (22±2°C). All the animals were born and raised in a 12-h-light/12-h-dark environment with an average illumination of 80 lx. Tap water and food pellets were provided. Mice were randomly divided into the DM group and the non-diabetes group and maintained on a high-fat and sugar diet composed of (by mg) 10% sugar, 10% lard, 5% yolk, 1% cholesterol, and 0.2% bile salt and a standard chow diet, respectively. Each mouse remained on the assigned diet throughout the whole experiment. After eight weeks, the DM group was intraperitoneally injected with 80 mg/kg STZ. The tail vein blood glucose was measured every three days two times after the injection, and those with blood glucose ≥16.7 mmol/L were considered DM mice [62–64]. The experiments were conducted between 10:00 and 14:00. STZ (Sigma) was dissolved in cold 50 mM citric acid buffer (pH 4.5).

### SF treatment

The mice were injected i.p. with 1.0 mg/kg SF (S8046, LKT Laboratories Inc., St. Paul, MN, USA) and/or 1.0 mg/kg PX12 for 2 weeks. The control mice were injected with PBS. All injections were performed at 10:00 am.

### Preparation of stretched retinal vessels

The retinal vasculature was labeled with lectin-BS (Sigma) for morphologic observation. Briefly, the eyeball was enucleated, fixed in 4% paraformaldehyde overnight at 4°C and then washed with PBS. Then, the entire retina was dissected from the eye cup, flat-mounted on a slide, and incubated overnight in PBS containing 0.2% Triton-X 100 and 20 μg/ml isolectin-BS. The slides were washed with PBS and incubated in PBS containing Alexa Green 488-conjugated streptavidin (Vector) concentration of 1:1000 for 2 h at 37°C. Then, the slides were washed with PBS and subsequently observed and photographed using a fluorescence microscope.

### Morphological analysis via quantitative histology

Quantitative histology was performed as described [33]. After sacrificing the mice via CO_2_ inhalation, the mouse eyes were enucleated at the indicated time points. The enucleated eyes were immersed in 4% paraformaldehyde containing 20% isopropanol, 2% trichloroacetic acid, and 2% zinc chloride for 24 h and then in 70% ethanol for 24-60 h. After alcohol dehydration, the eyes were embedded in paraffin, and 5-μm-thick sagittal sections containing the entire retina, including the optic disc, were sliced. The retinal sections were stained with hematoxylin-eosin (HE). In each of the superior and inferior hemispheres, we measured the thickness of different layers in the retina by the software (Nikon).

### Electroretinography (ERG)

ERG (GOTEC, China) was performed as previously described [45]. Briefly, the mice were kept in total darkness overnight after different treatments (control group, diabetes group, diabetes + SF group, diabetes+SF+PX12 group) before the ERG recording. After the mice were anesthetized and the pupils were dilated, full-field ERG at a light intensity of 10 cd·s/m^2^ and a band pass of 1–300 Hz was performed using a GUOTE ERG system. For the quantitative analysis, the A- and B-wave amplitudes were measured. The ERG waveforms of both eyes in the same animal were recorded simultaneously.

### Transfection

HUVEC cells were transfected with Trx shRNA plasmid or control shRNA plasmid (Dr. Hiroshi Masutani, Japan), which was performed with Lipofectamine2000 according to the manufacturer’s instructions. After transfection, the cells were treated with NG/HG to preparation for flow cytometry and cell viability.

The data are presented as the mean ± SD. The statistical analyses were performed using one-way analysis of variance (ANOVA) for continuous variables. SPSS version 17.0 was used for all of the statistical analyses. Statistical significance was defined as p<0.05.

## CONCLUSION

These findings indicate that diabetes can induce retina neuronal cell degeneration prior to endothelial damage, and Trx plays a key role in this process in cell culture and an animal model. This underlying mechanism may be related to activation of the Trx/ASK1/p-p38/Trx-interacting protein pathway.

## Abbreviations

Trx: thioredoxin
HG: high glucose
SF: sulforaphane
STZ: Streptozotocin
DR: diabetic retinopathy
Txnip: thioredoxin interacting protein
ERG: electroretinography
ONL: outer nuclear layer
INL: inner nuclear layer
RGC: retinal ganglion cell
MAPK: mitogen-activated protein kinase
MKKK: MAPK kinase kinase
ASK1: apoptosis signal-regulating kinase 1
HUVECs: human umbilical vein endothelial cells
HE: hematoxylin-eosin
